# Comparison of stress in implant-supported monolithic zirconia fixed partial dentures between canine guidance and group function occlusal patterns: A finite element analysis

**DOI:** 10.15171/joddd.2019.014

**Published:** 2019-08-14

**Authors:** Mahmood Robati Anaraki, Ali Torab, Taymaz Mounesi Rad

**Affiliations:** ^1^Dental and Periodontal Research Center, Department of Prosthodontics, Faculty of Dentistry, Tabriz University of Medical Science, Tabriz, Iran; ^2^Department of Prosthodontics, Faculty of Dentistry, Tabriz University of Medical Science, Tabriz, Iran

**Keywords:** Dental implants, finite element analysis, fixed partial denture, fracture strength, zirconia

## Abstract

***Background.*** Monolithic zirconia is an emerging material for crowns and bridges. The possibility of full digital design has made it an attractive alternative material for implant-supported prostheses. A proper design is vital in the success of such a prosthesis like any other. This study, in the shortage of scientific evidence, has tried to assess the stress distribution of occlusal forces inside the implant-prosthesis system of a 3-unit bridge made of monolithic zirconia.

***Methods.*** A 3-unit monolithic zirconia bridge supported by two implant fixtures placed on the teeth #13 and #15 was digitalized. It was converted to a mesh of 59000 nodes and 34000 elements. Five types of occlusal forces (one as vertical centric, two at 15º and 30º simulating canine pattern of lateral movement, and two at 15º and 30º simulating group function pattern) were applied. The stress distribution among all the components of the implant-bridge system was assessed using Ansys Workbench 14 software and finite element analysis.

***Results.*** The maximum stress was between 286 and 546 MPa, which were found in either the fixture‒abutment screw area or in the upper part of the pontic connector between the canine and first premolar. The maximum pressure increased with an increase in the angle of occlusal force. Significantly higher stress was recorded in the group function occlusal pattern.

***Conclusion.*** Monolithic zirconia can be promising in designing bridges in the canine‒premolar area. However, proper design is necessary with more attention to the connectors and types of occlusal forces.

## Introduction

Use of dental implants to replace missing or hopeless teeth has become widespread almost worldwide. They are used as single units to replace one tooth each, to support fixed partial dentures (FPDs) when two dental implants are used to cover three or four missed teeth, or to support removable dentures. Among them, the design for implant-supported FPDs is more controversial and seems to need more comprehensive studies.^1^

Although the high success rates of single-unit dental implants are highly documented,^2,3^ studies have shown that technical and biological complications can occur in almost one-third of implant-supported FPDs.^1^ Thus, more care should be taken in choosing a material and a design for such dental prostheses. Metal‒ceramic crowns and pontics supported by metal abutments (titanium or gold) are the most established choice of material at the moment. This is probably due to the wide availability of material and technology and relatively lower costs. However, zirconia-based full-ceramic materials (abutments and restorative crowns/bridges) have been introduced as an alternative to metal‒ceramic implant-supported FPDs.^4^

Although the metal‒ceramic FPDs are still routinely used,^4^ many advantages are claimed for zirconia-based and full-ceramic implant-supported crowns and bridges over the metal‒ceramic ones, especially when produced with a digital workflow by using the CAD-CAM system. Advantages of ceramic prostheses include, but not limited to, better esthetics, reduced possibility of gingival discoloration, greater translucency of the material, lower bacterial adhesion, better marginal integration between crowns and abutments, faster processing time, and uniform thickness of cementation space, which might result in more homogenous spread of force stress. The many claimed advantages of ceramic prostheses, if confirmed with scientific evidence, overcome its disadvantages such as the need for high technology, costs and brittleness of this type of material.^5,6^

Several ceramic materials have been tried for fabricating full-ceramic crowns and bridges. Each type has different physical and optical properties. However, when implants are used, the selection of these ceramics must be made accurately with extra care. Moreover, the FPD and tooth preparation design, insertion technique and adhesive luting agent should match the properties of the ceramic material. One more important thing to consider is the type of occlusion and the way occlusal forces are applied in the centric occlusion and lateral movements.^5,6^

Some ceramic materials like glass-reinforced, Al_2_O_3_-reinforced and feldspathic ceramics encountered some problems and therefore, have been deemed as not appropriate for implant-supported FPDs by many researchers.^7,8^ Other ceramic materials such as crystalline-dominated ceramics, and polycrystalline ceramics, meet the required properties for implant-supported FPDs to some extent and, therefore, are being used by clinicians. However, amongst all the different types of material used for full ceramic FPDs, zirconia-based ceramics have been the best known one and have attracted the attention of many researchers, clinicians and producers as a promising material. This is well true when implant-supported FPDs are designed for posterior areas, where strength becomes more important due to the size of occlusal forces in this region.^7-9^

The development of zirconia materials has continued and has proved promising to achieve better properties. For instance, better optical properties have been produced by changing particle size and distribution.^10,11^ One more recently introduced alternative to PFM and zirconia‒ceramic prosthesis is monolithic zirconia. The optical properties of zirconia are well improved, almost comparable to those of ceramics, while monolithic nature of the prosthesis minimizes the possibility of fracture or chipping and enhances its structural properties. On the other hand, it enables the technician to fabricate the whole prosthesis using CAD/CAM. Therefore, it seems that practically all the optical and mechanical properties that are needed to make a good FPD, especially in the posterior region, are achieved by monolithic zirconia crowns and bridges. The increasing use of this material worldwide can support the idea. In fact, many researchers and clinicians now recommend the use of monolithic zirconia in all types of fixed prostheses, including implant-supported FPDs and complete arches.^10,12^

In spite of all the good physical and mechanical properties of monolithic zirconia-based crowns and bridges used with implant supports, a variety of clinical failures have been reported. These failures have been due to problems in the framework, connector area, or excess forces transferred to the fixture's surrounding tissues. Therefore, being a relatively new material, more studies are necessary to better understand the case selection and optimal designs of this material in order to prevent or reduce the occurrence of these problems.^13,14^

One of the controversies over FPD design is around the type of occlusal relationship it should have with the opposing teeth (opposite dental arch). When it comes to the FPDs in canine and premolar area, choosing between a canine guidance and group function relationship becomes a matter, when the FPD is supported by real teeth or implants.^15^ Studies have shown that activities of facial muscles might change after replacement of missed teeth, and this change might be different in canine guidance and group function occlusal designs.^16^ This finding makes it important to choose between these two occlusal patterns from the beginning of treatment planning.

Some researchers claim that group function occlusal pattern in lateral movements of an FPD is associated with greater marginal bone loss around the implants. This can be due to the greater amount of occlusal stress exerted on the implant fixtures, or because of the angle of occlusal forces in lateral movements.^17^ Moreover, some claim that the possibility of contact with opposing teeth in non-functional lateral movements or non-working side contacts increases with a group function design.^17^ Despite the disadvantages mentioned, several studies can be found in favor of group function design, especially when the whole dental system has been taken into account instead of the FPD alone. A proper group function in lateral movements is hard to achieve, but if achieved, it is claimed to produce less attrition, better periodontal support and stronger chewing ability.^18,19^ Of course, there are some studies that have investigated some elements of the two occlusal types and found that they are equally good and equally acceptable in FPD design.^20,21^

The stress that is transferred from occlusal loads to each part of an implant-supported FPD has been studied by a limited number of research groups. Such investigation cannot be carried out properly in vivo. The best way would be the digital reproduction of an FPD with supporting implants and surrounding bone as a complex in a software program to analyze the effects of stimulated occlusal loads on each part and each corner of each part of the complex. Previous studies have used a 3D solid modeling software program such as SolidWorks Office, 2007 Version, and have used finite element analysis (FEA). The FEA uses a numerical technique to comprehensively quantify any analysis of a physical phenomenon: the distribution of a given force in solid materials and the material’s behavior in response to the force, in our case. Such a structural analysis allows the determination of stress and strain on layers of different materials, or body tissues, attached to one another resulting from an external force, thermal change, and other factors. This analysis is rather complicated. All the elements or materials are preferably assumed homogenous and isotropic and having linear elastic characteristics for this type of analysis.^22-24^

To the best of our knowledge no study has yet assessed the distribution of occlusal forces on different levels of an implant-supported FPD, comparing the canine guidance and group function lateral movement designs. This study can be one of the best in clearing up doubts about the two designs.

## Methods

A laboratory study using FEA was designed to assess the stress level applied to the partial fixed prosthetic appliances placed on implant fixtures, and to compare the amount and distribution of this stress when canine guidance and group function occlusal patterns are used. As mentioned above, the complex system of bearing occlusal stress along a fixed bridge was divided into smaller and simpler elements for better understanding and analysis, with the use of FEA method.

Creation of a 3-D model of the implant-bridge complex was one of the first challenges. A plastic model of upper dental arch with screw-driven plastic teeth (Nissin, Japan) was used for this purpose. The model had all the 16 upper teeth. A simple modeling technique has been used in many similar studies. However, as precise remodeling of the FPD was one of the key points to achieve accurate results, a detailed geometric method was used in the current study.

The plastic model and teeth were scanned to have a complete view of teeth and their sockets. The size and morphology of teeth were adjusted based on the measurements given by Stanley.^25^ Then the assembled solid model needed for this study, containing teeth #13 and #15 as abutments and tooth #14 as pontic (upper right canine and premolars), was produced by placing the teeth in their places in the model.

**Figure 1:**
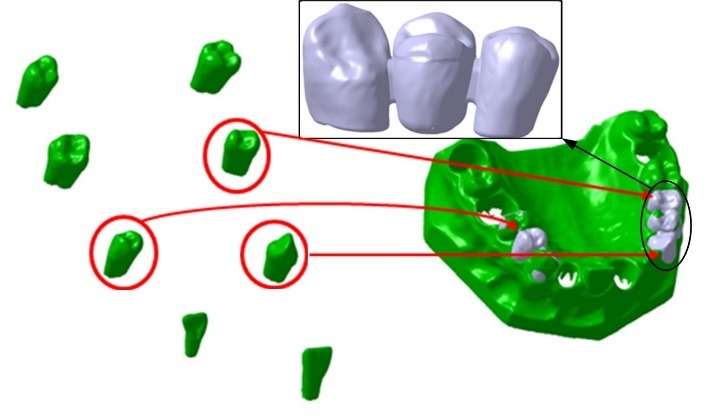
Volume model of teeth #13, #14 and #15 and their assembly on gingival cavities and modified bridge model.

A 3-D laser scanner was used. The scanner had a high accuracy with 0.01-mm resolution. The model, with 8 teeth mounted on it, and the separate eight other teeth, were all scanned and their dimensions were measured. The scanner’s output was in ‘clouding points’ format, which was stored and manipulated using Catia V5R14 software (Dassault System: UK, 2009).‏ Surface ‘Modeling’, ‘Solid Modeling’ and creation of final bridge model were conducted using the same software. This was processed and created by the clouding points file. The surface modeling picture of the model among a sample of a three-unit bridge which was created in the Catia environment is demonstrated in Figure 1.

Two titanium fixtures, measuring 5*12 mm each, were selected for the three-unit fixed bridge. Two identical abutments with a diameter of 4.5 mm, total length of 5.5 mm, total axial tapering of 6º and radial chamfer shoulder with 1-mm depth were also used. This marginal design type was chosen as it has been demonstrated to transmit less concentrated stress to the underlying structure in comparison to the other marginal designs.^26^ The exact geometry for implants and abutments was obtained from the 3D drawings of components provided by the implant system manufacturer (TBR: France, 2018) with 3D modelling software (CATIA V5R14, Dassault Systems: UK, 1998-2009).

The prosthesis of the 3-unit FPD was developed according to manufacturer’s instructions and guidelines on monolithic zirconia (Lava Plus: 3M ESPE, USA). Each unit of the prosthesis was connected to the adjacent one with a 4*4-mm connector. The pieces of the bridge were carefully assembled on the implant fixtures. The whole file was then exported to the FEA software. Ansys Workbench 14 software (AN- SYS Inc., Canonsburg, Pennsylvania, USA) was used for static nonlinear analysis of the stresses transferred to each point of the model bridge. The necessary material specifications of each part of the bridge were derived from manufacturers and were given to the Ansys Workbench 14 software. These are briefed in Table 1.

**Table 1:** Mechanical characteristics of materials used in the implant‒bridge system

**Material**	**Part**	**Density** **Kg mm** ^-3^	**Young's Modulus MPa**	**Poisson's Ratio**	**Bulk Modulus MPa**	**Shear Modulus MPa**
1	Implant	4.62e-006	1.15e+005	0.35	1.2778e+005	42593
2	Screw	4.62e-006	1.15e+005	0.36	1.369e+005	42279
3	Abutment	4.62e-006	1.15e+005	0.35	1.2778e+005	42593
4	Bone	1.85e-006	13000	0.3	10833	5000
5	Zirconia	5.7e-006	2.e+005	0.35	1.2222e+005	74074

The restoration was defined as cemented to the abutments with 0.025-mm-thick dual-cured resin cement (RelyX ARC, 3M ESPE AG, Seefeld, Germany). The type of connection between each two components of the bridge were defined as full coupling, based on the real connection types in a real implant-supported bridge for the FEA software. The degrees of freedom and possibility of displacement of connection nodes between the fixtures and bone were assumed as zero throughout the model.

**Figure 2:**
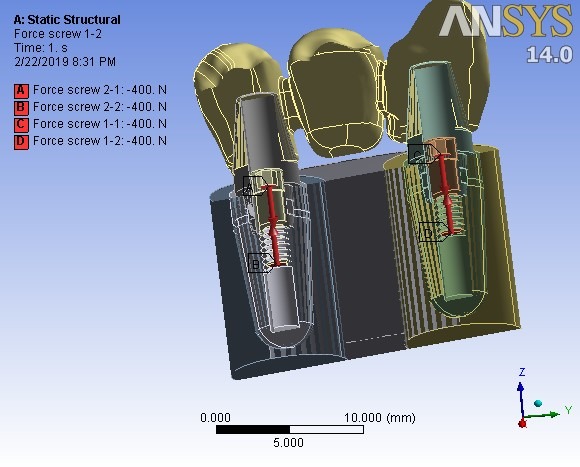
Pre-loading stage on assembled screw at 400 N.

**Figure 3:**
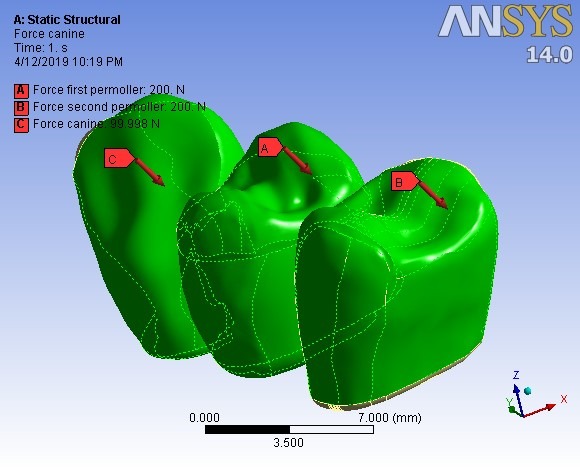
The stage of the axial force definition with a 30º angle on all the three teeth based on force type number 5.

A preloading force of 400 N (for screw fixation of abutments) was applied (Figure 2). Then five different types of loadings followed. First, the bridge was loaded as in the centric occlusion condition when masticatory forces are applied to all the teeth. In this loading, a force of 100 N was applied to the canine, and 200-N forces were applied to each premolar; all of them were vertically applied with zero degrees relative to the vertical line (Table 2). Then the loads were applied in two oblique directions of 15º and 30º in two occlusal movement conditions of canine guidance and group function. In the canine guidance condition, the oblique loads were applied to the canine only, while the loads were applied to all the three teeth in the group function position (Figure 3). The values of loads are given in Table 2.

**Table 2:** Configuring the types and amounts of loading on each tooth of the 3-unit bridge

	**Type of occlusion**	**Degree of force application**	**Force on tooth #13 (N)**	**Force on tooth #14 (N)**	**Force on tooth #15 (N)**
**1**	**Canine guidance**	15	100	--	--
**2**	**Canine guidance**	30	100	--	--
**3**	**Both**	0	100	200	200
**4**	**Group function**	15	100	200	200
**5**	**Group function**	30	100	200	200

**Figure 4:**
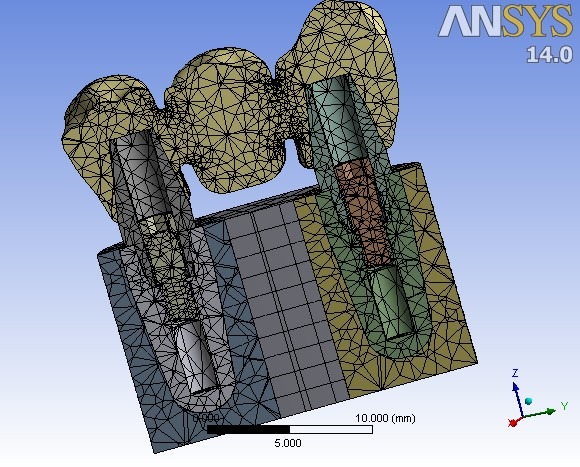
A cross-sectional view of the meshed implant‒bridge system.

The assembled complex, including the bridge, abutments, fixtures and the surrounding bone, was converted to a matrix or mesh of nodes and elements. The number of nodes and elements in the analysis were approximately 59000 and 34000, respectively (Figure 4). Displacement of each node after application of occlusal forces was monitored and considered to assess the distribution of stress. All the model materials were assumed to be homogeneous, with linearly elastic characteristics, and isotropic. Mechanical properties of material types are presented in Table 1. von-Mises equivalent stress criteria were used to study the distribution of external stress in different levels of the implant‒bridge complex.

## Results

As explained above, in this study finite element analysis was conducted on a digital design of a three-unit FPD of teeth #13, #14 and #15, which was made of monolithic zirconia placed on two implant fixtures‏ replacing roots of a canine and a second premolar. von-Mises equivalent stress was used as yield strength criteria.

Table 3 shows the amount and the location of the maximum stress exerted on the whole system and on the FPD by the five types of loading explained in Table 2. The high amount of stress was exerted on three points in all the five types of occlusal loads. Two points were located at the places in which abutments are screwed inside the fixtures and preloaded. The other high-stress point was the connector area between the canine and the first premolar. As shown in Table 2, the maximum stress area was located on abutment‒fixture screws, and not on the FPD in occlusal load types 1 and 2. However, in all the other three occlusal load types the highest stress bearing point among the whole implant‒bridge complex was located inside the FPD: the upper part of the connector between teeth #13 and #14.

**Table 3:** The maximum stress bearing amount and location on the whole implant-bridge system and on the FPD using Von-Mises criteria

**Force type (refer to Table 2)**	**Maximum stress on the whole system**		**Maximum stress in the bridge part**	
Order	Location	Amount (MPa)	Location	Amount (MPa)
1	Screws' body	286.0	The upper part of pontic between 13 and 14	27.9
2	Screws' body	297.1	The upper part of pontic between 13 and 14	81.9
3	The upper part of pontic between 13 and 14	286.2	The upper part of pontic between 13 and 14	286.2
4	The upper part of pontic between 13 and 14	369.6	The upper part of pontic between 13 and 14	369.6
5	The upper part of pontic between 13 and 14	546.0	The upper part of pontic between 13 and 14	546.0

**Figure 5:**
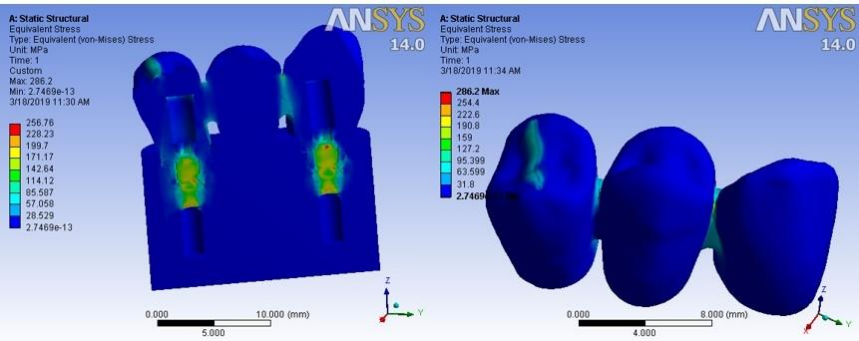
An example illustration of stress force exerted on the implant‒bridge system.

A cross-sectional view of the whole system, (a), and an outline view of the FPD, (b), in ‘clouding points’ format, while the occlusal force was applied, are illustrated in Figure 5 as an example. As the side bar shows, the areas in blue color were bearing the minimum stress, while the color change towards red means higher stress bearing. The illustrations showed that the maximum stress bearing areas were almost the same in all the five types of occlusal loads, but the measurements were different.

## Discussion

The current study successfully tried to analyze the force stress distribution in a complex system of implant-supported monolithic zirconia fixed partial denture in two different occlusal patterns of canine guidance and group function. As such investigation was not (and will not be) possible in vivo, digital modeling, FEA method and specific software programs were used.

It is rather difficult to create experimental models of dental prostheses and perform prototype experiments on them. It is not practically possible to create homological samples and in vivo or even in vitro tests are associated with errors. That is why FEA method is considered as an extremely effective alternative to performing applied studies on engineering issues and examining problems with dental prosthodontics. The validity of using FEA in the assessment of stress distribution in complex dental systems such as fixed prostheses has been shown in previous studies. The FEA allows researchers to accurately obtain the information they need from the subject matter and select the appropriate solutions to solve the problems.^27,28^

In the current study, using the same FEA method, which is a 3D comprehensive analysis, nonlinear loading of occlusal forces on the designed 3-unit FPD was carried out in zero, 15- and 30-degree angles. The maximum stress equivalent value for the angles of 0, 15 and 30 degrees was measured at 286, 370 and 546 MPa, respectively. The results indicated that increasing the angle of loading increased the maximum stress. Also, the maximum stress point in the bridge part of the system was found in the upper pontic area between the canine and the first premolar. The fracture strength of a zirconia bridge setup is approximately 900‒1200 MPa, which is far from the maximum tension obtained in the proposed models. Therefore, it can be concluded that such zirconia FPD is not susceptible to fracture in either occlusal patterns.

Previous studies have also found that the connector areas of an FPD are the weakest points and are the most prone area to fracture or deformation.^29^ A study by Kuroda et al^30^ found that the fracture resistance of a zirconia FPD could decrease from >900 MPa to <650 MPa by improper connector design. Therefore, it seems necessary to, bearing in mind the esthetic and hygienic considerations, increase the width and height of the connectors in terms of the length of the pontic. Of course, the amount of stress at this point was way below the fracture resistance of the material in the current study.

The points bearing the maximum stress did not change with a change in the angle of applied force in the current study. It happened probably because constraints and geometry remained the same. Therefore, the response of the structure remained the same. Some other studies that have used FEA for assessing the distribution of stress in FPDs have also reported that the stress points of the prosthesis, or the weakest link, did not change in their experiments with a change in the amount or angle of the occlusal stress.^22,30^ If the connector of an FDP was found as its weakest region, it remained as such, even when loading was shown to affect the general stress distribution pattern.^31,32^

The results of the current study showed that the maximum stress found in the group function occlusal pattern was notably higher than the canine guidance one. To justify such finding, it should be considered that although when forces are only applied to the canine, stress concentrations increases on the canine and the connective area between the canine and the premolar, the whole pressure decreases as less force is transferred via a single contact point. However, in the group function, due to the involvement of more closing muscles and also the proximity of the posterior teeth involved in the occlusion to the center of forces, destructive lateral forces in the long run cause much more force and presumably more wear and muscle fatigue and higher probability of material fracture.

## Conclusion

The highest stress was applied to the fixture‒abutment screw area and the upper part of the connector between canine retainer and premolar pontic. The force was measured between 286 MPa and 546 MPa, depending on the angle of occlusal force and occlusal pattern. Higher stress was found in the group function design. Monolithic zirconia bridge design showed overall fit for implant-supported FPDs. Proper connector design in the bridge part is recommended. Further detailed studies of similar nature but in different parts of the dental arch are suggested.

## Authors’ Contributions

MRA and AT contributed to the concept and design of the study, literature search, experimental studies, data acquisition and manuscript editing. TMR contributed to definition of intellectual content, literature search, experimental studies, data acquisition and manuscript preparation. All the authors have read and approved the final manuscript.

## Acknowledgements

This article was written based on a dataset from an MSc thesis entitled “Comparison of compressive strength, tensile strength, flexural strength and impact strength of rein-forced acrylic resin with TiO_2_ nanoparticles and conventional acrylic resin” registered at Tabriz University of Medical Sciences, Faculty of Dentistry (reference number 160/T). The thesis was supported by the Vice Chancellor for Research at Tabriz University of Medical Sciences.

## Funding

This study was supported by Vice Chancellor for Research (VCR), Faculty of Dentistry, Tabriz University of Medical Sciences (TUOMS), Tabriz, Iran.

## Competing Interests

The authors declare that they have no competing interest.

## Ethics Approval

Not applicable.
